# Supplementary material for: The Brain Protein Acylation System Responds to Seizures in the Rat Model of PTZ-Induced Epilepsy
Source: Int J Mol Sci. 2022 Oct 14;23(20):12302. doi: 10.3390/ijms232012302 (PMC9603846; doi:10.3390/ijms232012302)
Supplement: Supplementary file 1 [file ijms-23-12302-s001.zip › ijms-1917589-supplementary.pdf]

Supplementary Tables

Supplementary Table S1. List of detected acylation sites

Modification site	Peptide	Manual quantification
P02770|ALBU~K-Glu~588	K.AADK(+114.03)DNCFATEGPNLVAR.S	no
P04764|ENOA~Acety~89	K.KLNVVEQEK(+42.01)IDQLM(+15.99)IEM(+15.99)DGTENK.S	no
P04764|ENOA~K-Glu~89	K.KLNVVEQEK(+114.03)IDQLMIEMDGTENK.S	no
P04797|G3P~K-Glu~70	K.LVINGK(+114.03)PITIFQERDPANIK.W	no
P04797|G3P~K-Glu~117	K.RVIISAPSADAPMFVMGVNHEK(+114.03)YDNSLK.I	no
P04797|G3P~K-Glu~249	R.LEK(+114.03)PAKYDDIKK.V	no
P07335|KCRB~K-Glu~97	R.HGGYQPSDEHK(+114.03)TDLNPDNLQGGDDLDPNYVLSSR.V	no
P07335|KCRB~K-Glu~267	K.SK(+114.03)NYEFM(+15.99)WNPHLGYILTC(+57.02)PSNLGTGLR.A	no
P07335|KCRB~K-Mal~232	K.TFLVWINEEDHLRVISMQK(+86.00)GGNMK(+86.00)EVFTR.F	no
P0DMW0|HS71A~K-Suc~251	K.K(+100.02)DISQNK.R	no
P0DMW1|HS71B~K-Suc~251	K.K(+100.02)DISQNK.R	no
P10719|ATPB~K-Glu~133	K.VLDSGAPIK(+114.03)IPVGPETLGR.I	no
P10860|DHE3~K-Mal~90	K.LVEDLK(+86.00)TR.E	no
P16086|SPTN1~K-Glu~1952	K.MK(+114.03)GLNGK.V	no
P23565|AINX~K-Glu~216	K.K(+114.03)VESLLDELAFVR.Q	no
P34058|HS90B~K-Glu~427	K.ENYK(+114.03)K(+114.03)FYEAFSK.N	no
P46462|TERA~Acety~8	M.ASGADSK(+42.01)GDDLSTAILK.Q	no
P47709|RP3A~K-Mal~160	K.QVLPQPMPIK(+86.00)K.T	no
P47819|GFAP~K-Glu~187	R.K(+114.03)VESLEEEIQFLR.K	no
P47860|PFKAP~Acety~666	R.K(+42.01)N(+.98)VLGHMQQGGAPSPFDR.N	no
P48037|ANXA6~K-Glu~40	K.GFGSDK(+114.03)ESILELITSR.S	no
P53534|PYGB~Acety~3	M.AK(+42.01)PLTDSER.Q	no
P60905|DNJC5~K-Glu~46	K.YHPDK(+114.03)NPDNPEAADKFK.E	no
P61265|STX1B~K-Glu~125	R.K(+114.03)FVEVMTEYNATQSK.Y	no
P61765|STXB1~K-Glu~294	R.HK(+114.03)HIAEVSQEVTR.S	no
P61983|1433G~K-Glu~91	K.IEK(+114.03)ELEAVC(+57.02)QDVLSLLDNYLIK.N	no
P61983|1433G~K-Suc~78	K.IEMVRAYREK(+100.02)IEK.E	no
P62260|1433E~K-Glu~18	K.LAEQAERYDEMVESMK(+114.03)K.V	no
P62260|1433E~K-Glu~29	K.K(+114.03)VAGMDVELTVEER.N	no
P63018|HSP7C~K-Glu~328	K.LDK(+114.03)SQIHDIVLVGGSTR.I	no
P63018|HSP7C~K-Glu~512	R.LSK(+114.03)EDIER.M	no
P68035|ACTC~K-Glu~76	K.YPIEHGIITNWDDMEK(+114.03)IWHHTFYNELR.V	no
P68136|ACTS~K-Glu~76	K.YPIEHGIITNWDDMEK(+114.03)IWHHTFYNELR.V	no

P85845|FSCN1~K-Glu~250	K.VGK(+114.03)DELFALEQSCAQVVLQAANER.N	no
P85845|FSCN1~K-Glu~399	R.K(+114.03)VTGTLDANR.S	no
Q1WIM2|CADM2~K-Glu~396	R.HK(+114.03)GTYLTNEAK.G	no
Q3KR86|MIC60~K-Glu~234	R.TVEGALK(+114.03)ER.R	no
Q4FZU2|K2C6A~K-Glu~317	R.SLDLDSIIAEVK(+114.03)AQYEEIAK.R	no
Q4FZU2|K2C6A~K-Glu~415	R.GK(+114.03)LEGLEDALQK.A	no
Q561S0|NDUAA~K-Glu~181	R.K(+114.03)QC(+57.02)VDHYNEIKR.L	no
Q5XIF6|TBA4A~Acety~124	R.K(+42.01)LSDQC(+57.02)TGLQGFLVFHSFGGGTGSGFTSLLM(+15.99)ER.L	no
Q63537|SYN2~K-Glu~422	R.TPALSPQRPLTTQQPQSGTLK(+114.03)EPDSSK.T	no
Q63560|MAP6~K-Glu~254	R.TEGHEEK(+114.03)PLPPAQSQTQEGGPAAGK.A	no
Q66HF1|NDUS1~Acety~87	K.APK(+42.01)VVAAC(+57.02)AMPVMK.G	no
Q66HF1|NDUS1~K-Glu~471	K.K(+114.03)PMVVLGSSALQR.D	no
Q6IFV1|K1C14~K-Glu~180	K.TIEDLK(+114.03)SKILAATVDNANVLLQIDNAR.L	no
Q6IFV1|K1C14~K-Glu~240	R.ADLEMQIESLK(+114.03)EELAYLKK.N	no
Q6IFV1|K1C14~K-Glu~472	K.VMDVHDGK(+114.03)VVSTHEQVLR.T	no
Q6IG01|K2C1B~Acety~188	R.FLEQQNQVLQTK(+42.01)WELLQQVNTSTR.T	no
Q6IG02|K22E~K-Mal~413	K.EIK(+86.00)M(+15.99)EISELNR.T	no
Q6IMF3|K2C1~K-Glu~200	R.FLEQQNQVLQTK(+114.03)WELLQQVDTSTR.T	no
Q6P6Q2|K2C5~K-Glu~400	R.LRSEIDNVK(+114.03)K.Q	no
Q6P6V0|G6PI~K-Glu~524	K.K(+114.03)IEPELDGSSAVTSHDSSTNGLIGFIK.L	no
Q6PCU2|VATE1~K-Glu~69	K.K(+114.03)IQMSNLM(+15.99)NQAR.L	no
Q9ER34|ACON~K-Glu~144	R.AK(+114.03)DINQEVYNFLATAGAK.Y	no
Q9QUL6|NSF~K-Glu~462	K.ASTK(+114.03)VEVDMEKAESLQVTR.G	no
Q9QUL6|NSF~K-Glu~469	K.VEVDMEK(+114.03)AESLQVTR.G	no
Q9Z270|VAPA~K-Glu~211	K.VAHSDK(+114.03)PGSTSAVSFR.D	no
P13233|CN37~Acety~177	K.LK(+42.01)PGLEKDFLPLYFGWFLTK.K	yes
P23565|AINX~Acety~95	R.TNEK(+42.01)EQLQGLNDR.F	yes
P08461|ODP2~K-Glu~451	R.KELNK(+114.03)MLEGK.G	yes
P08461|ODP2~K-Mal~451	K.ELNK(+86.00)MLEGKGK.I	yes
P19527|NFL~Acety~340	K.QLQELEDK(+42.01)QNADISAM(+15.99)QDTINKLENELR.S	yes
P19527|NFL~K-Glu~303	R.AAK(+114.03)DEVSESRR.L	yes
P19527|NFL~K-Glu~340	K.QLQELEDK(+114.03)QNADISAMQDTINKLENELR.S	yes
P04636|MDHM~K-Suc~297	K(+100.02)GLEK	yes
P04642|LDHA~Acety~5	M.AALK(+42.01)DQLIVNLLK.E	yes
P04642|LDHA~K-Mal~155	K.ISGFPK(+86.00)NR.V	yes
P05708|HXK1~K-Glu~290	R.GSLNPGK(+114.03)QLFEK.M	yes
P07340|AT1B1~Acety~111	R.FLEK(+42.01)YK.D	yes

P07340|AT1B1~K-Glu~118	K.YKDSAQK(+114.03)DDMIFEDC(+57.02)GSMPSEPK.E	yes
P07340|AT1B1~K-Glu~173	K.EGK(+114.03)PC(+57.02)IIIK.L	yes
P07340|AT1B1~K-Glu~222	R.DEDK(+114.03)DKVGNIEYFGMGGFYGFPLQYYPYYGK.L	yes
P07340|AT1B1~K-Glu~224	R.DEDKDK(+114.03)VGNIEYFGMGGFYGFPLQYYPYYGK.L	yes
P09606|GLNA~K-Glu~107	R.K(+114.03)PAETNLR.H	yes
P09951|SYN1~K-Glu~179	R.SLK(+114.03)PDFVLIR.Q	yes
P09951|SYN1~K-Glu~281	K.VK(+114.03)VDNQHDFQDIASVVALTK.T	yes
P13233|CN37~K-Glu~177	K.LK(+114.03)PGLEK.D	yes
P13233|CN37~K-Glu~234	K.EK(+114.03)LDLVSYFGK.R	yes
P15865|H14~K-Suc~32	R.KAAGGAK(+100.02)R.K	yes
P17764|THIL~K-Glu~242	K.GK(+114.03)PDVVVKEDEEYKR.V	yes
P19332|TAU~K-Glu~592	K.K(+114.03)LDLSNVQSK.C	yes
P21707|SYT1~K-Glu~141	K.EEEK(+114.03)LGK.L	yes
P25113|PGAM1~K-Glu~225	K.NLK(+114.03)PIKPMQFLGDEETVR.K	yes
P25113|PGAM1~K-Glu~228	K.NLKPIK(+114.03)PMQFLGDEETVRK.A	yes
P32851|STX1A~K-Glu~126	R.K(+114.03)FVEVMSEYNATQSDYRER.C	yes
P35213|1433B~Acety~204	K.TAFDEAIAELDTLNEESYK(+42.01)DSTLIM(+15.99)QLLR.D	yes
P47942|DPYL2~K-Glu~472	R.K(+114.03)PFPDFVYKR.I	yes
P59215|GNAO~K-Glu~70	K.QYK(+114.03)PVVYSNTIQSLAAIVR.A	yes
P59215|GNAO~K-Mal~17	R.SK(+86.00)AIEKNLKEDGISAAK.D	yes
P62260|1433E~K-Suc~142	R.K(+100.02)EAAENSLVAYK.A	yes
P62630|EF1A1~K-Glu~273	R.VETGVLK(+114.03)PGMVVTFAPVNVTTEVK.S	yes
P63018|HSP7C~K-Suc~569	K.QK(+100.02)ILDK.C	yes
P63039|CH60~K-Suc~469	K.IGIEIIK(+100.02)R.A	yes
P63102|1433Z~Acety~202	K.TAFDEAIAELDTLSEESYK(+42.01)DSTLIMQLLR.D	yes
P63259|ACTG~K-Glu~215	K.EK(+114.03)LC(+57.02)YVALDFEQEMATAASSSSLEK.S	yes
Q05175|BASP1~K-Glu~9	K.KK(+114.03)KGYNVNDEK.A	yes
Q05175|BASP1~K-Glu~158	K.SDAAPAASDSK(+114.03)PSTEPAPSSK.E	yes
Q05962|ADT1~Acety~10	M.GDQALSFLK(+42.01)DFLAGGIAAAVSK.T	yes
Q09073|ADT2~K-Glu~245	R.K(+114.03)GTDIMYTGTLDC(+57.02)WR.K	yes
Q8VBU2|NDRG2~Acety~3	M.AELQEVQITEEK(+42.01)PLLPGQTPEAAK.E	yes

Supplementary Table S2. ANOVA analysis of the differences in acylations of the rat brain cortex proteins due to seizures and vitamins administration. Statistical significances of the group differences are estimated by 2-way ANOVA, followed by the multiple group comparison using the post-hoc Tukey's test.

Modification site	ANOVA factor p-values		Post-hoc Tukey p-values		
	seizures	vitamins	Ctrl-PTZ	Ctrl-Kin	Kin-PTZ
P13233|CN37~K-Glu~234	0,0004	0,0074	0,0077	0,0004	0,3353
P04642|LDHA~K-Mal~155	0,0006	0,3138	0,0004	0,0801	0,0505
P25113|PGAM1~K-Glu~228	0,0013	0,9750	0,0038	0,0034	0,9982

Q09073|ADT2~K-Glu~245	0,0020	0,8261	0,0062	0,0044	0,9888
Q05175|BASP1~K-Glu~158	0,0038	0,2669	0,0278	0,0037	0,6474
P47942|DPYL2~K-Glu~472	0,0067	0,9802	0,0051	0,1121	0,3816
P13233|CN37~K-Glu~177	0,0087	0,2674	0,0153	0,0277	0,9978
P04642|LDHA~Acety~5	0,0118	0,7668	0,0087	0,1357	0,3696
P08461|ODP2~K-Malo~451	0,0119	0,8443	0,0556	0,0132	0,7536
P25113|PGAM1~K-Glu~225	0,0171	0,8327	0,0257	0,0484	0,7955
P32851|STX1A~K-Glu~126	0,0203	0,5056	0,0463	0,0268	0,9393
P05708|HXK1~K-Glu~290	0,0228	0,3101	0,0629	0,0296	0,9341
P17764|THIL~K-Glu~242	0,0366	0,6500	0,0501	0,9951	0,0678
P19527|NFL~Glut_340	0,0447	0,5258	0,0511	0,1085	0,8888
P07340|AT1B1~K-Glu~118	0,0546	0,0595	0,9635	0,0688	0,1002
P09606|GLNA~K-Glu~107	0,0547	0,8204	0,3551	0,0435	0,4660
P35213|1433B~Acety~204	0,0635	0,1385	0,4760	0,0512	0,4093
P23565|AINX~Acet_95	0,0888	0,7764	0,4975	0,0748	0,5520
P63039|CH60~K-Suc~469	0,0928	0,8159	0,8341	0,0959	0,2463
P07340|AT1B1~K-Glu~224	0,0983	0,6968	0,0954	0,2383	0,8582
P19527|NFL~Glut_303	0,1008	0,5737	0,4001	0,6118	0,0877
P59215|GNAO~K-Mal~17	0,1101	0,0565	0,5226	0,0915	0,5042
P09951|SYN1~K-Glu~281	0,1706	0,5378	0,2140	0,9883	0,2527
P08461|ODP2~K-Glut~451	0,1759	0,4908	0,1699	0,3828	0,8858
P07340|AT1B1~K-Glu~222	0,2003	0,8083	0,1903	0,3961	0,8754
P15865|H14~K-Suc~32	0,2288	0,5858	0,2239	0,4052	0,9117
P63018|HSP7C~K-Suc~569	0,2516	0,8278	0,2223	0,7026	0,6438
P59215|GNAO~K-Glu~70	0,2901	0,8842	0,4017	0,3176	0,9767
Q8VBU2|NDRG2~Acety~3	0,3127	0,9510	0,5779	0,8468	0,2883
P19332|TAU~K-Glu~592	0,3249	0,2056	0,4297	0,3626	0,9912
P19527|NFL~Acet_340	0,3497	0,4920	0,9277	0,3610	0,5356
P04636|MDHM~K-Suc~297	0,3895	0,4183	0,6920	0,3571	0,8149
Q05962|ADT1~Acety~10	0,3915	0,7491	0,3686	0,8797	0,6817
P07340|AT1B1~K-Glu~173	0,4149	0,8205	0,7492	0,3819	0,7995
P09951|SYN1~K-Glu~179	0,5133	0,9320	0,5186	0,6560	0,9725
P62630|EF1A1~K-Glu~273	0,5687	0,6179	0,5431	0,7888	0,9194
Q05175|BASP1~K-Glu~9	0,5826	0,9434	0,5561	0,8144	0,9214
P62260|1433E~K-Suc~142	0,6405	0,7399	0,6700	0,9969	0,7157
P63102|1433Z~Acety~202	0,7048	0,3079	0,8432	0,6946	0,9749
P07340|AT1B1~Acety~111	0,7074	0,8445	0,7596	0,7354	0,9990
P63259|ACTG~K-Glu~215	0,7348	0,1947	0,7127	0,9148	0,9167
P21707|SYT1~K-Glu~141	0,7921	0,7910	0,8675	0,9888	0,7927
P13233|CN37~K-Acet~177	0,9632	0,1882	0,9770	0,9978	0,9632

Supplementary Table S3. Sample sizes of all measured parameters and their pairwise correlations after exclusion of missing data and outliers.

	PDH activity	OGDH activity	SIRT2	OADH	SIRT3	NAD, nmol/g	SIRT5	P62260|1433E~K-Suc~142	P63018|HSP7C~K-Suc~569	P05708|HXK1~K-Glu~290	P09951|SYN1~K-Glu~179	P19332|TAU~K-Glu~592	P21707|SYT1~K-Glu~141	Q09073|ADT2~K-Glu~245	P07340|AT1B1~Acety~111	P07340|AT1B1~K-Glu~173	P07340|AT1B1~K-Glu~222	P07340|AT1B1~K-Glu~224	P08461|ODP2~K-Glu~451	P59215|GNAO~K-Mal~17	P08461|ODP2~K-Mal~451	P15865|H14~K-Suc~32	P09951|SYN1~K-Glu~281	P63259|ACTG~K-Glu~215	Q8VBU2|NDRG2~Acety~3	P09606|GLNA~K-Glu~107	Q05175|BASP1~K-Glu~158	P62630|EF1A1~K-Glu~273	P59215|GNAO~K-Glu~70	P13233|CN37~Acety~177	P04636|MDHM~K-Suc~297	P63102|1433Z~Acety~202	P04642|LDHA~K-Mal~155	P63039|CH60~K-Suc~469	P32851|STX1A~K-Glu~126	Q05962|ADT1~Acety~10	Q05175|BASP1~K-Glu~9	P07340|AT1B1~K-Glu~118	P13233|CN37~K-Glu~234	P17764|THIL~K-Glu~242	P04642|LDHA~Acety~5	P25113|PGAM1~K-Glu~228	P47942|DPYL2~K-Glu~472	P13233|CN37~K-Glu~177	P35213|1433B~Acety~204	P19527|NFL~K-Glu~340	P25113|PGAM1~K-Glu~225	P19527|NFL~K-Glu~303	P23565|AINX~Acety~95	P19527|NFL~Acety~340
PDH activity	30	30	29	28	28	28	27	30	30	30	30	30	30	30	29	29	29	29	29	29	29	29	29	29	29	29	29	28	28	28	28	28	27	27	27	27	27	27	27	27	26	26	26	26	25	25	25	24	19	18
OGDH activity	30	30	29	28	28	28	27	30	30	30	30	30	30	30	29	29	29	29	29	29	29	29	29	29	29	29	29	28	28	28	28	28	27	27	27	27	27	27	27	27	26	26	26	26	25	25	25	24	19	18
SIRT2	29	29	29	27	27	27	26	29	29	29	29	29	29	29	28	28	28	28	28	28	28	28	28	28	28	28	28	28	27	27	27	27	26	26	27	26	26	26	26	26	26	26	26	25	24	25	25	24	18	18
OADH	28	28	27	28	26	26	26	28	28	28	28	28	28	28	27	27	27	27	27	27	27	28	28	27	27	27	27	26	26	26	26	26	25	25	25	25	25	27	25	25	24	24	24	25	24	23	23	23	18	17
SIRT3	28	28	27	26	28	27	25	28	28	28	28	28	28	28	27	27	27	27	27	27	27	27	27	27	27	27	27	26	26	26	26	26	25	25	25	25	26	25	26	25	24	24	24	24	23	23	23	23	17	17
NAD, nmol/g	28	28	27	26	27	28	25	28	28	28	28	28	28	28	27	27	27	27	27	27	27	27	27	27	27	27	27	26	26	26	26	26	25	25	25	25	25	25	25	25	24	24	24	24	23	24	23	22	17	16
SIRT5	27	27	26	26	25	25	27	27	27	27	27	27	27	27	26	26	26	26	26	26	26	26	26	26	26	26	26	25	26	25	26	25	24	24	24	24	24	25	24	25	23	23	23	24	23	22	22	22	18	15
P21707|SYT1~K-Glu~141	30	30	29	28	28	28	27	30	30	30	30	30	30	30	29	29	29	29	29	29	29	29	29	29	29	29	29	28	28	28	28	28	27	27	27	27	27	27	27	27	26	26	26	26	25	25	25	24	19	18
P63018|HSP7C~K-Suc~569	30	30	29	28	28	28	27	30	30	30	30	30	30	30	29	29	29	29	29	29	29	29	29	29	29	29	29	28	28	28	28	28	27	27	27	27	27	27	27	27	26	26	26	26	25	25	25	24	19	18
P62260|1433E~K-Suc~142	30	30	29	28	28	28	27	30	30	30	30	30	30	30	29	29	29	29	29	29	29	29	29	29	29	29	29	28	28	28	28	28	27	27	27	27	27	27	27	27	26	26	26	26	25	25	25	24	19	18
P09951|SYN1~K-Glu~179	30	30	29	28	28	28	27	30	30	30	30	30	30	30	29	29	29	29	29	29	29	29	29	29	29	29	29	28	28	28	28	28	27	27	27	27	27	27	27	27	26	26	26	26	25	25	25	24	19	18
P05708|HXK1~K-Glu~290	30	30	29	28	28	28	27	30	30	30	30	30	30	30	29	29	29	29	29	29	29	29	29	29	29	29	29	28	28	28	28	28	27	27	27	27	27	27	27	27	26	26	26	26	25	25	25	24	19	18
Q09073|ADT2~K-Glu~245	30	30	29	28	28	28	27	30	30	30	30	30	30	30	29	29	29	29	29	29	29	29	29	29	29	29	29	28	28	28	28	28	27	27	27	27	27	27	27	27	26	26	26	26	25	25	25	24	19	18
P19332|TAU~K-Glu~592	30	30	29	28	28	28	27	30	30	30	30	30	30	30	29	29	29	29	29	29	29	29	29	29	29	29	29	28	28	28	28	28	27	27	27	27	27	27	27	27	26	26	26	26	25	25	25	24	19	18
P07340|AT1B1~Acety~111	29	29	28	27	27	27	26	29	29	29	29	29	29	29	29	29	29	29	28	28	28	28	28	28	28	28	28	27	27	27	27	27	27	27	26	27	26	26	26	26	26	25	25	25	25	24	24	23	18	18
P07340|AT1B1~K-Glu~224	29	29	28	27	27	27	26	29	29	29	29	29	29	29	29	29	29	29	28	28	28	28	28	28	28	28	28	27	27	27	27	27	27	27	26	27	26	26	26	26	26	25	25	25	25	24	24	23	18	18
P07340|AT1B1~K-Glu~222	29	29	28	27	27	27	26	29	29	29	29	29	29	29	29	29	29	29	28	28	28	28	28	28	28	28	28	27	27	27	27	27	27	27	26	27	26	26	26	26	26	25	25	25	25	24	24	23	18	18
P07340|AT1B1~K-Glu~173	29	29	28	27	27	27	26	29	29	29	29	29	29	29	29	29	29	29	28	28	28	28	28	28	28	28	28	27	27	27	27	27	27	27	26	27	26	26	26	26	26	25	25	25	25	24	24	23	18	18
P08461|ODP2~K-Glu~451	29	29	28	27	27	27	26	29	29	29	29	29	29	29	28	28	28	28	29	29	28	28	28	28	28	28	28	28	28	27	27	27	26	26	26	26	26	26	26	26	26	25	25	25	25	25	24	23	18	18
P59215|GNAO~K-Mal~17	29	29	28	27	27	27	26	29	29	29	29	29	29	29	28	28	28	28	29	29	28	28	28	28	28	28	28	28	28	27	27	27	26	26	26	26	26	26	26	26	26	25	25	25	25	25	24	23	18	18
P08461|ODP2~K-Mal~451	29	29	28	27	27	27	26	29	29	29	29	29	29	29	28	28	28	28	28	28	29	28	28	28	28	28	28	27	27	27	27	27	26	26	26	26	27	26	26	26	25	26	26	26	24	24	24	24	18	17
P09951|SYN1~K-Glu~281	29	29	28	28	27	27	26	29	29	29	29	29	29	29	28	28	28	28	28	28	28	29	29	28	28	28	28	27	27	27	27	27	26	26	26	26	26	27	26	26	25	25	25	25	24	24	24	23	18	18
P15865|H14~K-Suc~32	29	29	28	28	27	27	26	29	29	29	29	29	29	29	28	28	28	28	28	28	28	29	29	28	28	28	28	27	27	27	27	27	26	26	26	26	26	27	26	26	25	25	25	25	24	24	24	23	18	18
P63259|ACTG~K-Glu~215	29	29	28	27	27	27	26	29	29	29	29	29	29	29	28	28	28	28	28	28	28	28	28	29	28	28	28	27	27	27	27	27	26	27	26	26	27	26	27	26	25	25	25	25	24	24	24	23	18	18
Q8VBU2|NDRG2~Acety~3	29	29	28	27	27	27	26	29	29	29	29	29	29	29	28	28	28	28	28	28	28	28	28	28	29	28	28	27	27	27	27	27	26	26	26	26	26	26	26	27	25	25	25	25	24	25	24	23	19	17
P09606|GLNA~K-Glu~107	29	29	28	27	27	27	26	29	29	29	29	29	29	29	28	28	28	28	28	28	28	28	28	28	28	29	29	27	27	27	27	27	26	26	26	26	26	26	26	26	25	25	25	25	24	24	24	24	18	17
Q05175|BASP1~K-Glu~158	29	29	28	27	27	27	26	29	29	29	29	29	29	29	28	28	28	28	28	28	28	28	28	28	28	29	29	27	27	27	27	27	26	26	26	26	26	26	26	26	25	25	25	25	24	24	24	24	18	17
P62630|EF1A1~K-Glu~273	28	28	28	26	26	26	25	28	28	28	28	28	28	28	27	27	27	27	28	28	27	27	27	27	27	27	27	28	27	26	26	26	25	25	26	25	25	25	25	25	26	25	25	24	24	25	24	23	17	18
P59215|GNAO~K-Glu~70	28	28	27	26	26	26	26	28	28	28	28	28	28	28	27	27	27	27	28	28	27	27	27	27	27	27	27	27	28	26	27	26	25	25	25	25	25	25	25	25	25	24	24	24	24	24	23	22	18	17
P13233|CN37~Acety~177	28	28	27	26	26	26	25	28	28	28	28	28	28	28	27	27	27	27	27	27	27	27	27	27	27	27	27	26	26	28	26	26	25	25	26	26	25	25	25	26	24	25	24	25	23	23	24	22	17	18
P04636|MDHM~K-Suc~297	28	28	27	26	26	26	26	28	28	28	28	28	28	28	27	27	27	27	27	27	27	27	27	27	27	27	27	26	27	26	28	26	26	25	25	25	25	25	25	25	25	24	24	24	23	23	23	22	19	16
P63102|1433Z~Acety~202	28	28	27	26	26	26	25	28	28	28	28	28	28	28	27	27	27	27	27	27	27	27	27	27	27	27	27	26	26	26	26	28	26	25	25	25	25	25	25	25	24	24	24	24	24	23	23	22	18	16

P04642|LDHA~K-Mal~155	27	27	26	25	25	25	24	27	27	27	27	27	27	27	27	27	27	27	26	26	26	26	26	26	26	26	26	25	25	25	26	26	27	25	24	25	24	24	24	24	25	23	23	23	24	22	22	21	18	16
P63039|CH60~K-Suc~469	27	27	26	25	25	25	24	27	27	27	27	27	27	27	27	27	27	27	26	26	26	26	26	27	26	26	26	25	25	25	25	25	25	27	24	25	25	24	25	24	24	23	23	23	23	22	22	21	17	17
Q05962|ADT1~Acety~10	27	27	26	25	25	25	24	27	27	27	27	27	27	27	27	27	27	27	26	26	26	26	26	26	26	26	26	25	25	26	25	25	25	25	25	27	24	24	24	24	24	23	23	24	23	22	22	21	17	17
P32851|STX1A~K-Glu~126	27	27	27	25	25	25	24	27	27	27	27	27	27	27	26	26	26	26	26	26	26	26	26	26	26	26	26	26	25	26	25	25	24	24	27	25	24	24	24	24	24	24	24	24	22	23	23	22	16	18
Q05175|BASP1~K-Glu~9	27	27	26	25	26	25	24	27	27	27	27	27	27	27	26	26	26	26	26	26	27	26	26	27	26	26	26	25	25	25	25	25	24	25	24	24	27	24	26	24	23	24	24	24	22	22	22	23	16	17
P07340|AT1B1~K-Glu~118	27	27	26	27	25	25	25	27	27	27	27	27	27	27	26	26	26	26	26	26	26	27	27	26	26	26	26	25	25	25	25	25	24	24	24	24	24	27	24	24	23	23	23	24	23	22	22	22	17	16
P13233|CN37~K-Glu~234	27	27	26	25	26	25	24	27	27	27	27	27	27	27	26	26	26	26	26	26	26	26	26	27	26	26	26	25	25	25	25	25	24	25	24	24	26	24	27	24	23	23	23	23	22	22	22	22	17	17
P17764|THIL~K-Glu~242	27	27	26	25	25	25	25	27	27	27	27	27	27	27	26	26	26	26	26	26	26	26	26	26	27	26	26	25	25	26	25	25	24	24	24	24	24	24	24	27	23	24	23	23	22	23	23	21	17	16
P04642|LDHA~Acety~5	26	26	26	24	24	24	23	26	26	26	26	26	26	26	26	26	26	26	26	26	25	25	25	25	25	25	25	26	25	24	25	24	25	24	24	24	23	23	23	23	26	23	23	22	23	23	22	21	16	17
P25113|PGAM1~K-Glu~228	26	26	26	24	24	24	23	26	26	26	26	26	26	26	25	25	25	25	25	25	26	25	25	25	25	25	25	25	24	25	24	24	23	23	24	23	24	23	23	24	23	26	24	23	21	22	24	23	15	17
P13233|CN37~K-Glu~177	26	26	25	25	24	24	24	26	26	26	26	26	26	26	25	25	25	25	25	25	26	25	25	25	25	25	25	24	24	25	24	24	23	23	24	24	24	24	23	23	22	23	24	26	22	21	22	22	16	16
P47942|DPYL2~K-Glu~472	26	26	26	24	24	24	23	26	26	26	26	26	26	26	25	25	25	25	25	25	26	25	25	25	25	25	25	25	24	24	24	24	23	23	24	23	24	23	23	23	23	24	26	24	21	22	23	22	16	16
P35213|1433B~Acety~204	25	25	24	24	23	23	23	25	25	25	25	25	25	25	25	25	25	25	25	25	24	24	24	24	24	24	24	24	24	23	23	24	24	23	22	23	22	23	22	22	23	21	21	22	25	21	20	20	17	15
P19527|NFL~K-Glu~340	25	25	25	23	23	24	22	25	25	25	25	25	25	25	24	24	24	24	25	25	24	24	24	24	25	24	24	25	24	23	23	23	22	22	23	22	22	22	22	23	23	22	22	21	21	25	22	20	16	16
P25113|PGAM1~K-Glu~225	25	25	25	23	23	23	22	25	25	25	25	25	25	25	24	24	24	24	24	24	24	24	24	24	24	24	24	24	23	24	23	23	22	22	23	22	22	22	22	23	22	24	23	22	20	22	25	21	15	18
P19527|NFL~K-Glu~303	24	24	24	23	23	22	22	24	24	24	24	24	24	24	23	23	23	23	23	23	24	23	23	23	23	24	24	23	22	22	22	22	21	21	22	21	23	22	22	21	21	23	22	22	20	20	21	24	14	15
P23565|AINX~Acety~95	19	19	18	18	17	17	18	19	19	19	19	19	19	19	18	18	18	18	18	18	18	18	18	18	19	18	18	17	18	17	19	18	18	17	16	17	16	17	17	17	16	15	16	16	17	16	15	14	19	8
P19527|NFL~Acety~340	18	18	18	17	17	16	15	18	18	18	18	18	18	18	18	18	18	18	18	18	17	18	18	18	17	17	17	18	17	18	16	16	16	17	18	17	17	16	17	16	17	17	16	16	15	16	18	15	8	18

Supplementary Table S4. Gene-disease associations of acylated proteins which are quantified in this study, obtained from DisGeNET database (v7.0). Only gene-disease associations belonging to class "Nervous System Diseases" with gda score more than 0.5 are shown. Proteins containing acylations significantly changed upon seizures (Figure 1) are marked in grey.

Gene	Gene_id	Disease	Disease_id	Type	Disease_Class	Semantic_Type	N_genes_d	N_SNPs_d	Score_gda	EL_gda	EI_gda	N_PMIDs	N_SNPs_gda	First_Ref	Last_Ref
NEFL	4747	CHARCOT-MARIE-TOOTH DISEASE, AXONAL, TYPE 2E (disorder)	C1843225	disease	Congenital, Hereditary, and Neonatal Diseases and Abnormalities; Nervous System Diseases	Disease or Syndrome	1	16	0.95		1.0	24	16	2000	2019
HSPD1	3329	Spastic paraplegia 13, autosomal dominant	C1854467	disease	Congenital, Hereditary, and Neonatal Diseases and Abnormalities; Nervous System Diseases	Disease or Syndrome	1	2	0.92		1.0	6	2	1975	2016
MAPT	4137	Pick Disease of the Brain	C0236642	disease	Nervous System Diseases; Mental Disorders	Disease or Syndrome	213	83	0.8		0.975	243	28	1998	2020
MAPT	4137	Frontotemporal dementia	C0338451	disease	Nutritional and Metabolic Diseases; Nervous System Diseases; Mental Disorders	Disease or Syndrome	320	215	0.8		0.978	460	43	1997	2020
HSPD1	3329	Leukodystrophy, Hypomyelinating, 4	C2677109	disease	Congenital, Hereditary, and Neonatal Diseases and Abnormalities; Nutritional and Metabolic Diseases; Nervous System Diseases	Disease or Syndrome	3	2	0.73		1.0	7	1	2002	2018
MAPT	4137	Supranuclear Palsy, Progressive, 1, Atypical	C1850077	disease	Pathological Conditions, Signs and Symptoms; Eye Diseases; Nervous System Diseases	Disease or Syndrome	1	4	0.71		1.0	1	4	2007	2007
ACTG1	71	Deafness, Autosomal Dominant 20	C1858172	disease	Pathological Conditions, Signs and Symptoms; Nervous System Diseases; Otorhinolaryngologic Diseases	Disease or Syndrome	1	10	0.7	strong	1.0	8	10	1996	2014
SLC25A4	291	Progressive External Ophthalmoplegia with Mitochondrial DNA Deletions, Autosomal Dominant, 2	C1836460	disease	Pathological Conditions, Signs and Symptoms; Nutritional and Metabolic Diseases; Eye Diseases; Musculoskeletal Diseases; Nervous System Diseases	Disease or Syndrome	1	4	0.7	strong	1.0	9	4	2000	2017
DLAT	1737	Pyruvate Dehydrogenase E2 Deficiency	C1855565	disease	Congenital, Hereditary, and Neonatal Diseases and Abnormalities; Nutritional and Metabolic Diseases; Nervous System Diseases	Disease or Syndrome	1	2	0.7		1.0	4	2	2005	2016
HK1	3098	Neuropathy, hereditary motor and sensory, Russe type	C1854449	disease	Congenital, Hereditary, and Neonatal Diseases and Abnormalities; Nervous System Diseases	Disease or Syndrome	1	3	0.7	strong	1.0	6	3	2000	2017
MAPT	4137	Alzheimer's Disease	C0002395	disease	Nervous System Diseases; Mental Disorders	Disease or Syndrome	3397	1843	0.7		0.99	997	23	1988	2020
MAPT	4137	Progressive supranuclear palsy	C0038868	disease	Pathological Conditions, Signs and Symptoms; Eye Diseases; Nervous System Diseases	Disease or Syndrome	176	52	0.7		0.953	149	10	1995	2019

MAPT	4137	Parkinsonian Disorders	C0242422	group	Nervous System Diseases	Disease or Syndrome	373	95	0.7		1.0	101	11	1998	2019
MAPT	4137	Supranuclear Palsy, Progressive, 1	C4551863	disease	Pathological Conditions, Signs and Symptoms; Eye Diseases; Nervous System Diseases	Disease or Syndrome	6	6	0.7		1.0	9	6	1999	2014
NEFL	4747	Charcot-Marie-Tooth disease, demyelinating, Type 1F	C1843164	disease	Congenital, Hereditary, and Neonatal Diseases and Abnormalities; Nervous System Diseases	Disease or Syndrome	1	6	0.7		1.0	4	6	2000	2013
SYN1	6853	Epilepsy, X-Linked, with Variable Learning Disabilities and Behavior Disorders	C1845343	disease	Pathological Conditions, Signs and Symptoms; Congenital, Hereditary, and Neonatal Diseases and Abnormalities; Nervous System Diseases; Mental Disorders	Disease or Syndrome	1	7	0.7		1.0	2	7	2004	2011
MAPT	4137	Tauopathies	C0949664	group	Nervous System Diseases	Disease or Syndrome	245	43	0.6		0.994	359	14	1999	2020
YWHAE	7531	Miller Dieker syndrome	C0265219	disease	Congenital, Hereditary, and Neonatal Diseases and Abnormalities; Nervous System Diseases	Disease or Syndrome	182	9	0.6		1.0	11	0	2000	2017
MAPT	4137	Semantic Dementia	C0338462	disease	Nutritional and Metabolic Diseases; Nervous System Diseases; Mental Disorders	Mental or Behavioral Dysfunction	20	4	0.56		1.0	13	2	1998	2017
GNAO1	2775	Early infantile epileptic encephalopathy with suppression bursts	C0393706	disease	Nervous System Diseases	Disease or Syndrome	81	10	0.51	definitive	1.0	13	0	1997	2019
GNAO1	2775	Movement Disorders	C0026650	group	Nervous System Diseases	Disease or Syndrome	362	247	0.5	definitive	1.0	20	3	1997	2019
MAPT	4137	Memory Disorders	C0025261	disease	Pathological Conditions, Signs and Symptoms; Nervous System Diseases; Behavior and Behavior Mechanisms	Mental or Behavioral Dysfunction	62	0	0.5		1.0	6	0	2002	2018
MAPT	4137	Parkinson Disease	C0030567	disease	Nervous System Diseases	Disease or Syndrome	2078	990	0.5		0.972	142	40	1999	2019
MAPT	4137	Memory impairment	C0233794	phenotype	Pathological Conditions, Signs and Symptoms; Nervous System Diseases; Behavior and Behavior Mechanisms	Mental or Behavioral Dysfunction	763	48	0.5		1.0	15	4	2002	2019
MAPT	4137	Dementia	C0497327	disease	Nervous System Diseases; Mental Disorders	Mental or Behavioral Dysfunction	816	176	0.5		0.992	127	13	1995	2020

Supplementary Figures

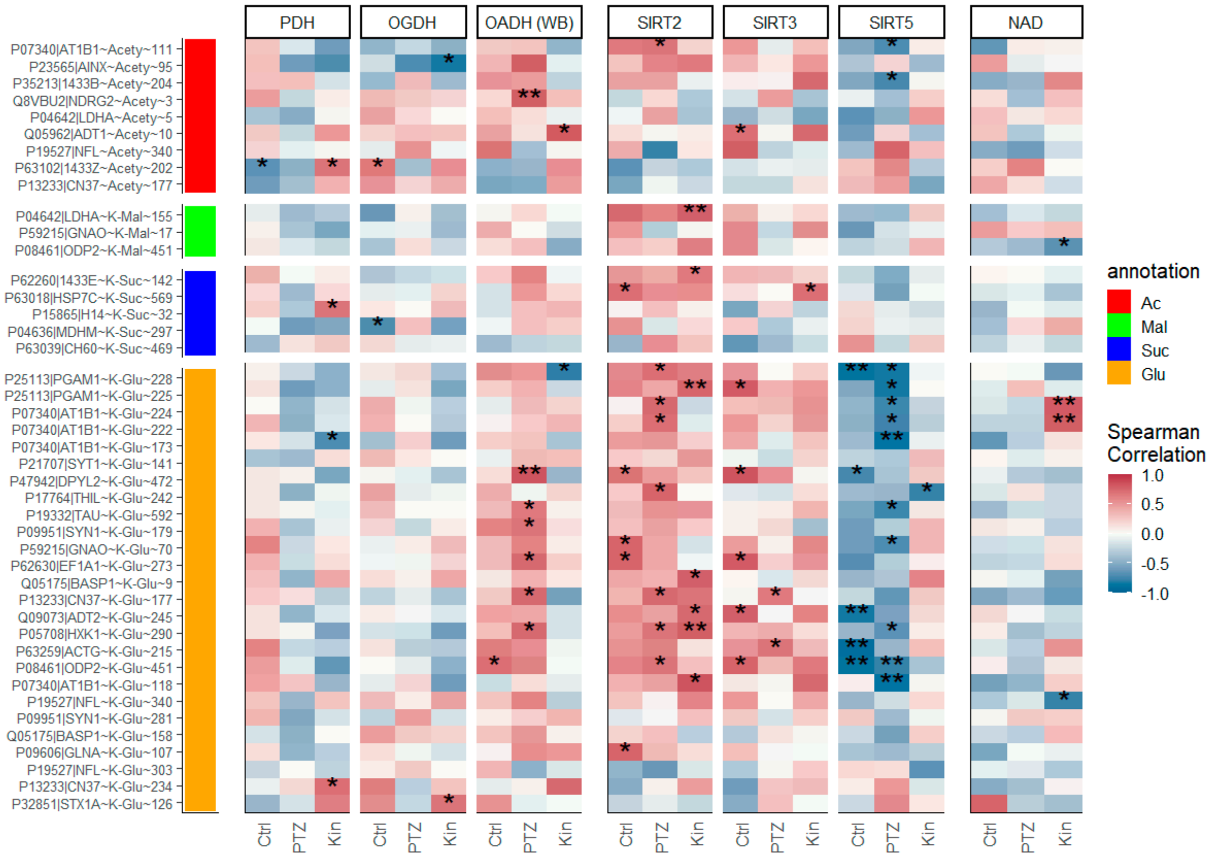

Supplementary Figure S1. Groupwise Spearman correlations of acylations with the activity of 2-oxo acid dehydrogenases, the level of NAD+ and sirtuins 2, 3 and 5.

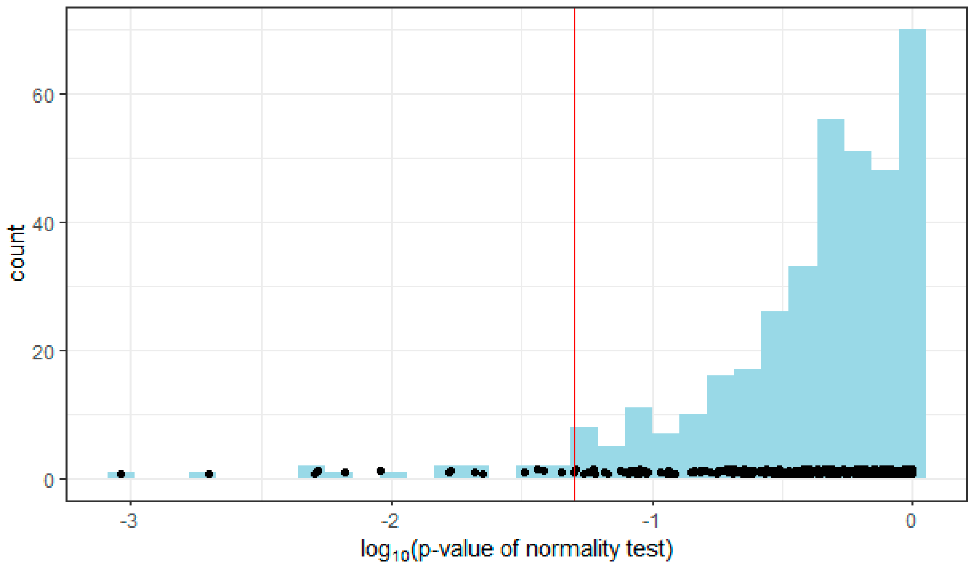

Supplementary Figure S2. Histogram of Shapiro-Wilk test for normality p-values in all groups of all measured parameters. Red line indicate p=o.o5 treshhold.
